# Índice de Desenvolvimento Humano e Doenças Crônicas no Brasil entre 1980 e 2019

**DOI:** 10.36660/abc.20230213

**Published:** 2023-04-27

**Authors:** Alfredo José Mansur, Lucia Pereira Barroso

**Affiliations:** 1 Hospital das Clínicas Faculdade de Medicina Universidade de São Paulo São Paulo SP Brasil Instituto do Coração do Hospital das Clínicas da Faculdade de Medicina da Universidade de São Paulo, São Paulo SP – Brasil; 2 Universidade de São Paulo Instituto de Matemática e Estatística São Paulo SP Brasil Universidade de São Paulo Instituto de Matemática e Estatística, São Paulo, SP – Brasil

**Keywords:** Doença Crônica/epidemiologia, Desenvolvimento Humano, Expectativa de Vida/tendências, Marginalização Social, Desemprego, Pobreza, Atenção Primária à Saúde

O índice de desenvolvimento humano sintetiza dimensões das sociedades humanas expressas em expectativa de vida, educação e o padrão de vida da população. A expectativa de vida seria uma expressão das condições de saúde da população. A educação seria expressa pelos anos de escolaridade. O padrão de vida seria expresso pela renda *per capita* . Admite-se também que o índice não abarca todas as dimensões relevantes da experiência da condição humana nas sociedades e contribui por aproximar-se de variáveis indicadoras. Ainda que muito útil, o índice de desenvolvimento humano é reconhecido como não abrangente - por exemplo, escapam ao índice as dimensões de desigualdade, pobreza, insegurança, empoderamento.^1 , 2^ Isto posto, é sempre oportuno para a comunidade médico-científica ter em mente as dimensões examinadas no índice de desenvolvimento humano e desenvolver estudos recorrentes e atualizações no decorrer do tempo, pois dinâmicas são as condições nas quais vivem sociedades humanas.

No estudo ora apresentado,^3^ os autores examinaram o índice de desenvolvimento humano no Brasil no decorrer de quatro décadas, com base nos dados disponibilizados pelo DATASUS com emprego de séries temporais com taxas de mortalidade (causa básica de óbito) por doenças crônicas não transmissíveis padronizadas por 100.000 habitantes divididos por quartis em cada unidade da Federação brasileira. No cômputo global houve redução das taxas de mortalidade padronizadas de mortalidade por 100.000 habitantes em todas as faixas etárias por doenças do aparelho circulatório. De modo interessante, a atualização trazida pelo estudo dos autores indica que a participação das doenças crônico-degenerativas, ainda que com redução no âmbito nacional geral, não se verificou de modo homogêneo em todas as unidades da Federação. Na Tabela 1 foram apresentados os índices de mortalidade que revelaram ter diminuído a participação dessas doenças em estados da região Sudeste, Sul e Centro-Oeste, diferentemente da participação que se verificou elevar-se em outras regiões do país.

Desse modo os autores fizeram uma contribuição que pode ser mais um dado a auxiliar as diferentes instâncias voltadas para a prevenção e o tratamento das doenças crônico-degenerativas não transmissíveis. Na Figura 4 demonstrou-se que quanto maior foi o índice de desenvolvimento humano a variação da mortalidade por doenças crônico-degenerativas foi menor. A redução foi maior nas unidades da Federação com índice de desenvolvimento humano maior ou igual a 0,7. Associaram essa evolução à melhoria das condições socioeconômicas, um dos pilares – ainda que não o único – do índice de desenvolvimento humano.

Mais recentemente outras variáveis foram incluídas como objeto de estudo no âmbito do desenvolvimento humano e nas suas relações com a saúde a respeito dos determinantes sociais da saúde: gradiente social, estresse, cuidados na infância, exclusão social, trabalho, desemprego, suporte social, dependência química, alimentação, transporte.^4 , 5^ Diferenças foram relatadas em experiência de outro país quanto a etnia e foram consideradas oportunidades de contínuo trabalho.^6^ Mais recentemente depois de seguimento médico de mediana de 23,6 anos (intervalo interquartil 19,6 anos – 28,9 anos) as comorbidades múltiplas foram identificadas em participantes hígidos há 50 anos como mecanismo de influência na mortalidade, mesmo em países com acesso universal ao atendimento de cuidados com a saúde no sistema público.^7^ Em outro estudo, a diferença entre a expectativa de vida entre estados da mesma federação também demonstrou variação ainda que pudesse haver elevação de renda;^6 , 8^ essa variação foi observada particularmente na população de menor renda, causada por doenças cardíacas ou câncer. A população de renda mais alta obteve expectativa de vida semelhante. Foi sugerido que essas ocorrências se deveriam mais a comportamento (tabagismo, obesidade, sedentarismo) do que acesso a serviços de saúde, diferenças de moradia ou índice de desenvolvimento humano (índice GINI). Os autores formularam a hipótese de que fatores locais contribuiriam para a natureza do comportamento social e exposição a fatores de risco à saúde.^6^

Dessa forma temos mais um estudo de dados brasileiros a demonstrar que os cuidados à saúde dão oportunidade a abordagem ampla em variáveis que podem ser convergentes e aditivas do ponto de vista de prevenção e tratamento de doenças que conforme sugeridos na literatura podem também sofrer influência de variáveis relacionadas à educação, renda per capita, gradiente social, estresse, cuidados na infância, exclusão social, trabalho, desemprego, suporte social, dependência química, alimentação, transporte, entre outras variáveis possíveis.^4 , 5^
