# Supplementary material for: Índice de Desenvolvimento Humano e Doenças Crônicas no Brasil entre 1980 e 2019
Source: Arq Bras Cardiol. 2023 Apr 27;120(4):e20230213. [Article in Portuguese] doi: 10.36660/abc.20230213 (PMC10263422; doi:10.36660/abc.20230213)
Supplement: Translation

# Translation: Human Development Index and Chronic Diseases in Brazil between 1980 and 2019

**Authors:** Alfredo José Mansur, Lucia Pereira Barroso

**Affiliations:** 1 Brazil Instituto do Coração do Hospital das Clínicas da Faculdade de Medicina da Universidade de São Paulo, São Paulo SP – Brazil; 2 Brazil Universidade de São Paulo Instituto de Matemática e Estatística, São Paulo, SP – Brazil

**Keywords:** Chronic Disease/epidemiology, Human Development, Life Expectancy/trends, Social Marginalization, Unemployment, Poverty, Primary Health Care

The human development index synthesizes dimensions of human societies taking into consideration life expectancy at birth, education, and standard of living. Life expectancy is an expression of the health conditions of a particular population. Education is expressed as years of schooling. The standard of living is indicated by gross national income per capita. It is recognized that the human development index does not encompass every social determinant of the experience of citizens of a specific human society. Nonetheless, it is useful and a recognized methodological approach for measurements and comparisons; however, inequalities, poverty, human security, empowerment, and other characteristics may be missed.^1 , 2^That said, it is always opportune for the medical-scientific community to bear in mind the dimensions examined in the human development index and to develop recurrent studies and updates over time, as the conditions in which human societies live are dynamic.

In the current study,^3^the authors evaluated the human development index in Brazil over four decades (1980-2019). The data (the underlying cause of death) were retrieved from death certificates in a Unified Health System (SUS) public database relative to chronic non-infectious diseases with time series analyses. Mortality was corrected for 100.000 inhabitants distributed in quartiles in each Brazilian state.

There was an overall decrease in mortality rate through ages for circulatory system diseases. Interestingly, the update of this study demonstrated that despite an overall decrease in mortality rate from a national perspective, this decrease was not homogeneous in the different states. In Table 1, the mortality rates revealed that the participation of these diseases in the Southeast, South, and Middle-west; in other states, the participation of chronic degenerative non-infectious diseases increased.

The authors’ contribution is one more step in preventing and treating chronic degenerative non-infectious diseases. Figure 4 demonstrates that the higher the human development index, the lower mortality due to chronic degenerative diseases. The decrease was higher in the states with a human development index higher the 0.7. They inferred that this observation resulted from better socioeconomic conditions, one of the pillars of the human development index.

Additional variables were addressed as relevant for human development in relationship with health conditions considered social determinants of health: social gradient, stress, early infancy, social exclusion, work conditions, unemployment, social support, addiction, nutrition, and transportation.^4 , 5^Significant differences were reported in other experiences for ethnic issues and regarded as an opportunity for improvement.^6^

Recently, multiple co-morbidities were identified as a significant influence on mortality after a median of 23.6 years of follow-up (interquartile interval 19.6 years – 28.9 years) participants healthy 50 years before in a country with universal access to care in the public health system.^7^In another study, differences between life expectations in states of the same countries were reported despite increases in financial incomes;^6 , 8^the difference was more noticeable in lower income strata due to heart disease and cancer. Higher financial income strata demonstrated similar life expectancy. It was suggested that these findings might be due more to lifestyles (smoking, obesity, sedentarism) than to access to health care, housing, or human development (GINI). The authors hypothesized that local environmental factors might contribute to unhealthy life styles.^6^

This is one more study of Brazilian databases to demonstrate that in health care delivery, we may have opportunities for a broad view of socioeconomic dimensions that may add each other and converge to better prevention and treatment of chronic diseases through variables recently reemphasized such as education, gross national product per capita, social gradient, stress, early infancy care, social exclusion, work, unemployment, social support, addiction, diet, transportation among other potentially associated variables.^4 , 5^
